# Impaired Sensitivity to Thyroid Hormones Is Associated With Elevated Blood Glucose in Coronary Heart Disease

**DOI:** 10.3389/fendo.2022.895843

**Published:** 2022-06-15

**Authors:** Lu Yu, Zhu Li, Rongrong Yang, Guangwei Pan, Qi Cheng, Yuanyuan He, Yijia Liu, Fanfan Liu, Mei Ma, Tong Yang, Yang Wang, Jinyu Su, Yanchao Zheng, Shan Gao, Qiang Xu, Lin Li, Chunquan Yu

**Affiliations:** ^1^Tianjin University of Traditional Chinese Medicine, Tianjin, China; ^2^Second Teaching Hospital of Tianjin University of Traditional Chinese Medicine, Tianjin, China

**Keywords:** elevated blood glucose, coronary heart disease, thyroid hormone sensitivity, diabetes, prediabetes, resistance to thyroid hormones

## Abstract

**Context:**

Thyroid hormones influence glucose homeostasis through central and peripheral regulation. To date, the association between thyroid hormone sensitivity and elevated blood glucose (EBG) in patients with coronary heart disease (CHD) remains unknown. The purpose of this study was to investigate the association between thyroid hormone sensitivity and risk of EBG in patients with CHD, and to further explore their association in different sexes and ages.

**Methods:**

This large multicenter retrospective study included 30,244 patients with CHD (aged 30–80 years) between 1 January 2014 and 30 September 2020. Parameters representing central and peripheral sensitivity to thyroid hormones were calculated. Central sensitivity to thyroid hormones was assessed by calculating the Thyroid Feedback Quantile-based Index (TFQI), Thyroid-stimulating Hormone Index (TSHI), and Thyrotropin Thyroxine Resistance Index (TT4RI), and Parametric Thyroid Feedback Quantile-based Index (PTFQI); peripheral sensitivity to thyroid hormones was evaluated using the ratio of free triiodothyronine (FT3) /free thyroxine (FT4). Taking normal glucose tolerance (NGT) as a reference, logistic regression was used to analyse the relationship between central and peripheral thyroid hormone sensitivity and EBG in patients with CHD.

**Results:**

Among the 30,244 participants, 15,493 (51.23%) had EBG. The risk of EBG was negatively correlated with TSHI (OR: 0.91; 95%CI: 0.91 to 0.92; *P* < 0.001), TT4RI (OR: 0.99; 95% CI: 0.99 to 0.99; *P*<0.001), TFQI (OR: 0.82; 95%CI: 0.80 to 0.84; *P <*0.001) and PTFQI (OR: 0.76; 95%CI: 0.74 to 0.78; *P*<0.001). Compared to males and patients aged 60 and below, the OR value for EBG was lower in females and in patients aged over 60 years old. Conversely, EBG risk was positively associated with FT3/FT4 (OR: 1.08; 95% CI: 1.07 to 1.09; *P <*0.001) and in the sex-categorized subgroups, males had higher OR values than females.

**Conclusions:**

This study showed that thyroid hormone sensitivity is significantly associated with EBG in patients with CHD. This association is higher in females than in males, and the association in those aged over 60 years old is higher than that in patients aged 60 years and below.

**Graphical Abstract:**
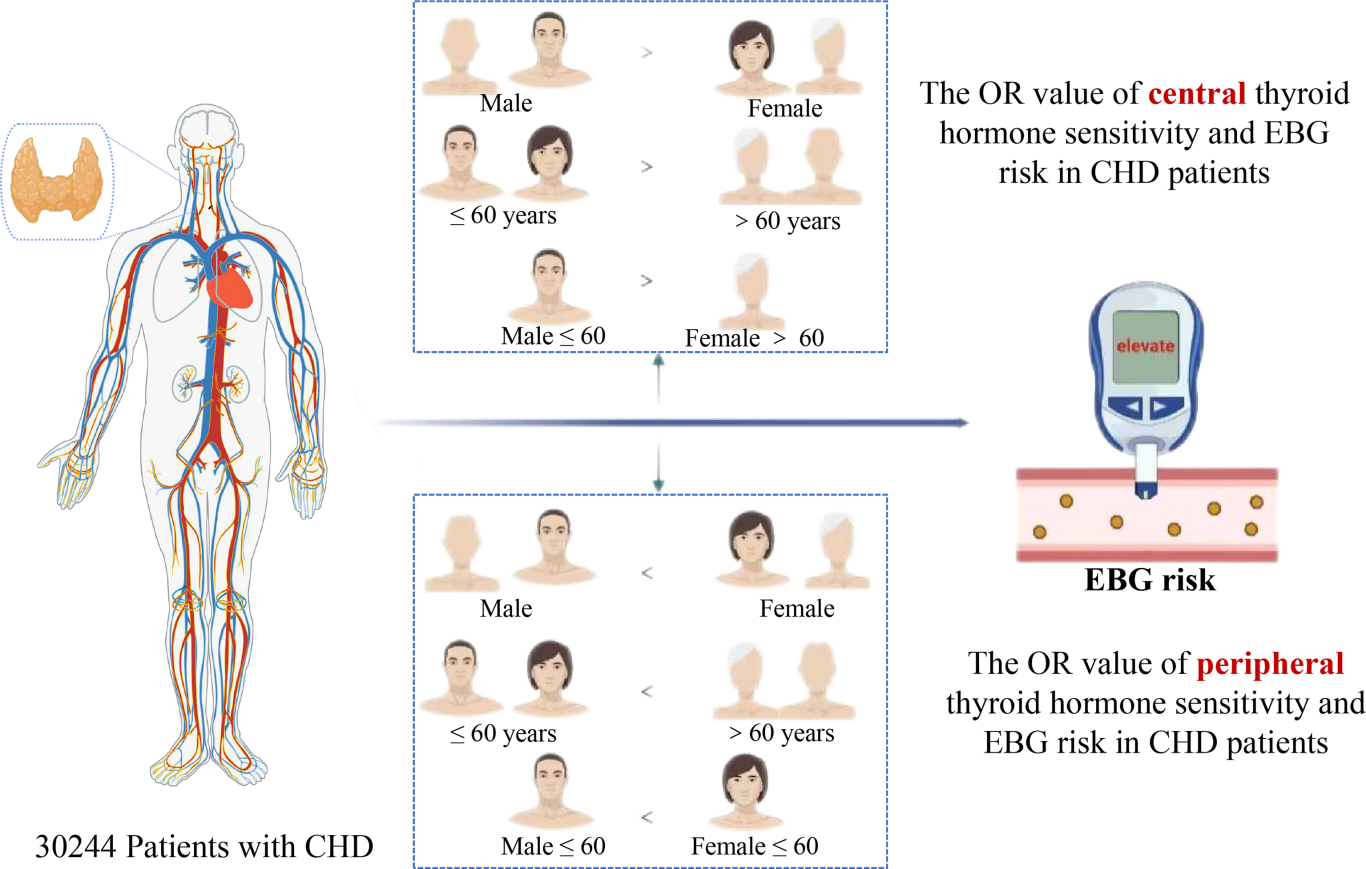
Images are from Biorender.com.

## Introduction

Cardiovascular diseases are the leading cause of death worldwide (1), seriously affecting the patient’s quality of life and longevity (2, 3). The vast majority of cardiac deaths are caused by coronary heart disease (CHD) secondary to coronary atherosclerosis (4). The relationship between CHD and thyroid hormones is very close, and abnormal thyroid hormones often accompany patients with CHD (5–9). In addition, patients with CHD and EBG are at a higher risk of nephropathy and other cardiovascular diseases. CHD complicated with diabetes is associated with decreased insulin sensitivity, impaired blood pressure regulation, damaged vascular endothelial cells and dysfunction of the fibrinolytic system (10, 11). Lowering blood sugar is extremely important to improve prognosis and prevent the onset of cardiovascular diseases (12–14). Diabetes has been clinically defined as fasting or postprandial hyperglycaemia or abnormally increased glucose excursion in response to an established glucose load. However, this clinical definition defines a relatively late stage in the disease process. Indeed, a defect in glucose homeostasis can be detected long before diabetes occurs.

For early prevention and treatment of secondary elevated blood glucose (EBG) in patients with CHD, identifying the risk factors of prediabetes and diabetes is a critical step. Recent theories show that hypothyroidism and subclinical hypothyroidism are risk factors for diabetes (15). However, some researchers believe that elevated thyroid stimulating hormone (TSH) is a risk factor for diabetes, and an increase in free triiodothyronine (FT3) and free thyroxine (FT4) has a protective effect on the occurrence of diabetes (16). TI de Vries et al. reported that there was no significant relationship between plasma TSH levels in the normal range and the incidence of diabetes in patients at high cardiovascular risk (17). These contradictory results are common. Moreover, almost all previous analyses focused on the influence of TSH and FT4 levels on the risk of prediabetes or diabetes. No research has investigated the association between thyroid hormone sensitivity and EBG in patients with CHD. Furthermore, results of previous studies investigating EBG prevalence in different sexes and ages have been inconsistent (18, 19). Due to inconsistent results, the relationship among EBG, sex, and age remains controversial.

The thyroid hormones regulate glucose homeostasis by interacting with the entire central nervous system and surrounding target organs (20). Therefore, this large-scale, multicenter retrospective study aimed to investigate the association of central and peripheral sensitivity to thyroid hormones in patients with CHD and EBG. Further, we aimed to explore the differences of these associations in different sexes and ages to provide a basis for clinical adjustments of medication according to the situation of individual patients with CHD, and to improve patient conditions.

## Methods

### Patients

**Figure 1:**
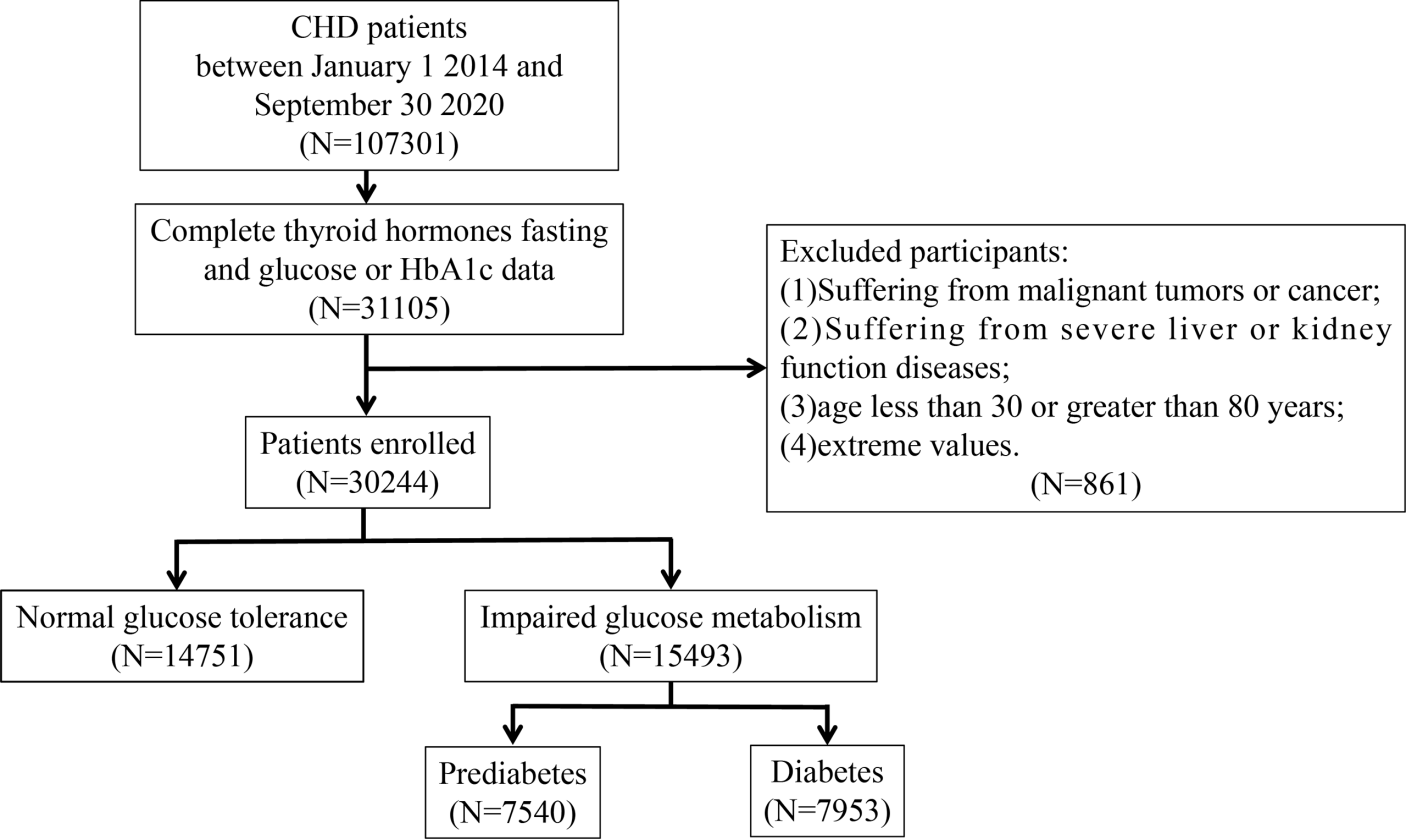
Flowchart of patient recruitment.

Participants in this study were 107,301 CHD inpatients of cardiology departments from 1 January 2014 to 30 September 2020 from six hospitals in Tianjin. Patients who were younger than 30 years or older than 80 years, had malignancy, infectious or severe liver or kidney disease, incorrect data, and lack of data on TSH, FT3, FT4, fasting blood glucose (FBG), or glycated haemoglobin (HbA1c) were excluded based on the study design. Ultimately, 30,244 participants were included in the study. A flowchart of the patient recruitment process is shown in **Figure 1**. This study was approved by the ethics committee of Tianjin University of Traditional Chinese Medicine (approval number TJUTCM-EC20190008) and registered with the Chinese Clinical Trial Registry on 14 July 2019 (registration number ChiCTR-1900024535) and ClinicalTrials.gov on 18 July 2019 (registration number NCT04026724).

### Data Collection

Trained medical staff collected personal medical history records. These records included information such as age, sex, history of smoking and drinking, which were investigated using standard structured questionnaires (21). Systolic blood pressure (SBP) and diastolic blood pressure (DBP) were measured by experienced technicians using automatic blood pressure monitors. Fasting venous blood samples were collected from all participants in the morning. FBG, total cholesterol (TC), high-density lipoprotein cholesterol (HDL-C), triglycerides (TG), low-density lipoprotein cholesterol (LDL-C), and HbA1c were measured directly using an automatic haematology analyser. Quality control was conducted in the laboratory according to standard procedures.

Hypertension is defined as elevated blood pressure (SBP ≥ 140 mmHg or DBP ≥ 90 mmHg) (22). Dyslipidaemia is defined as TG ≥ 1.7 mmol/L or TC ≥ 5.2 mmol/L or LDL ≥ 3.4 mmol/L or HDL ≤ 1.0 mmol/L (23). Different levels of glucose metabolism, namely normal glucose tolerance (NGT), prediabetes and diabetes, are defined as FBG < 5.6 mmol/L or HbA1c < 5.7%, FBG 5.6 to 6.9 mmol/L or HbA1c 5.7 to 6.4%, and FBG ≥ 7.0 mmol/L or HbA1c ≥ 6.5% (24). Participants with prediabetes or diabetes were assigned to the EBG group and the rest of the participants were assigned to the NGT group (25, 26).

### Indices of Thyroid Hormone Sensitivity

Central indices of thyroid hormone sensitivity included the TSH index (TSHI), TSH T4 resistance index (TT4RI), thyroid feedback quantile-based index (TFQI), and parametric thyroid feedback quantile-based index (PTFQI). TSHI was calculated as TSH (mIU/L) + 0.1345 * FT4 (pmol/L) (27). TT4RI was calculated as FT4 (pmol/L) * TSH (mIU/L) (28). TFQI and PTFQI were calculated using the algorithm developed by Laclaustra et al. (29) FT3/FT4 ratio was calculated to evaluate the peripheral thyroid hormone sensitivity.

### Statistical Analyses

The characteristics of the participants in the different groups were compared using χ2 (Chi-square) and Mann-Whitney tests. Age, sex, smoking, drinking, hypertension, and hyperlipidaemia were considered as potential confounding factors. The TSHI index was divided into tertiles: T1, TSHI index ≤ 1.5; T2: 1.50 < TSHI index < 2.49; and T3: TSHI index ≥ 2.49. The TT4RI index was divided into tertiles: T1: TT4RI index ≤ 9.01; T2: 9.01 < TT4RI index < 25.58; and T3: TT4RI index ≥ 25.58. The TFQI index was divided into tertiles: T1: TFQI index ≤ -0.23; T2: -0.23 < TFQI index < 0.12; and T3: TFQI index ≥ 0.12. The PTFQI index was divided into tertiles: T1, PTFQI index ≤ -0.13; T2: -0.13 < PTFQI index < 0.24; and T3: PTFQI index ≥ 0.24. The FT3/FT4 index was divided into tertiles: T1: FT3/FT4 index ≤ 0.29; T2: 0.29 < FT3/FT4 index < 0.41; and T3: FT3/FT4 index ≥ 0.41. Logistic regression analysis was used to compare the relationship between thyroid hormone sensitivity and EBG in the different tertiles. Missing values were calculated using the multiple-complement calculation method. All statistical analyses were performed using SPSS 24.0 (IBM Corp., New York, NY, USA).

## Results

### Baseline Characteristics

The baseline characteristics of the 30,244 participants are shown in **Table 1**. The average age of the subjects was 64 years old, and the proportion of females (48.3%) was slightly lower than that of males (51.7%). Among the participants, 15,493 patients demonstrated EBG (51.2%). The subjects were divided into two groups according to glucose metabolism level. Compared with NGT participants, EBG participants were more likely to be older females with hypertension, dyslipidaemia, and a tendency to smoke and drink. Hypertension, dyslipidaemia, age, smoking, and alcohol consumption were positively associated with EBG, and participants with EBG tended to have higher levels of FT3/FT4 and lower levels of TSHI, TT4RI, and PTFQI.

**Table 1:** Baseline characteristics.

Characteristic	Total (n = 30244)	NGT (n = 14751)	EBG (n = 15493)	*P*-value
Sex, n (%)				0.192
Males	15640 (51.71)	7605 (51.56)	8035 (51.86)	
Females	14604 (48.29)	7146 (48.44)	7458 (48.14)	
Age (y), median (IQR)	64 (58,69)	64 (57,68)	64 (58,69)	< 0.001
Dyslipidemia, %	20801 (68.78)	9346 (63.36)	11455 (73.94)	< 0.001
Hypertension, %	14686 (48.56)	6400 (43.39)	8286 (53.48)	< 0.001
Smoking, %	12535 (41.45)	5892 (39.94)	6643 (42.88)	< 0.001
Drinking, %	8872 (29.33)	4159 (28.19)	4713 (30.42)	< 0.001
FPG (mmol/L), median (IQR)	5.53 (4.90,6.96)	4.90 (4.56,5.20)	6.98 (6.1,8.89)	< 0.001
current antihypertensive medication, %	11851 (39.18)	4050 (27.46)	7801 (50.35)	< 0.001
current antilipidemic medication, %	10516 (34.77)	3416 (23.16)	7100 (45.83)	< 0.001
HbA1c (%), median (IQR)	6.30 (5.70,7.40)	5.70 (5.40,6.10)	6.7 (5.9,7.8)	< 0.001
FT3 (pmol/L), median (IQR)	3.83 (2.87,4.43)	3.99 (3.31,4.52)	3.61 (2.64,4.32)	< 0.001
FT4 (pmol/L), median (IQR)	12.05 (4.01,14.32)	12.16 (8.34,14.04)	11.88 (1.13,14.64)	< 0.001
TSH (mIU/L), median (IQR)	1.80 (1.18,2.75)	1.83 (1.21,2.78)	1.78 (1.14,2.72)	< 0.001
FT3/FT4, median (IQR)	0.33 (0.27,2.10)	0.33 (0.28,0.44)	0.34 (0.27,2.30)	< 0.001
TSHI, median (IQR)	2.06 (1.15,2.71)	2.16 (1.40,2.73)	1.93 (0.95,2.69)	< 0.001
TT4RI, median (IQR)	17.17 (4.55,30.91)	19.22 (8.50,32.44)	14.47 (2.76,29.44)	< 0.001
TFQI, median (IQR)	-0.05 (-0.33,0.22)	-0.02 (-0.29,0.22)	-0.08 (-0.37,0.21)	< 0.001
PTFQI, median (IQR)	0.06 (-0.24,0.32)	0.10 (-0.17,0.33)	0.01 (-0.31,0.31)	< 0.001

Data are presented as median (interquartile) or number (proportion, %).

### Relationship Between Thyroid Hormone Sensitivity and EBG

The association between thyroid hormone sensitivity and EBG was estimated by using different logistic regression models (**Table 2**) After adjusted for sex and age, as a continuous variable, TSHI, TT4RI, PTFQI, and TFQI levels were negatively associated, and FT3/FT4 levels was positively associated with EBG risk. These associations remained significant after multivariate adjustment. In the adjusted model, the OR values of TSHI, TT4RI, and PTFQI were the lowest at T3 when T1 was used as a reference. In addition, when used as a continuous variable, FT3/FT4 level was positively correlated with EBG. Considering the differences between prediabetes and diabetes in the presence of abnormal glucose metabolism, different stratified analyses were performed (**Supplementary Tables S1–S3**).

**Table 2:** Relationship between thyroid hormone sensitivity and EBG.

Variables	EBG			
	OR (95% CI)^1^	*P*-value	OR (95% CI)^2^	*P*-value
TSHI	0.86 (0.85-0.87)	< 0.001	0.91 (0.91-0.92)	< 0.001
T1	Reference		Reference	
T2	0.56 (0.55,0.57)	< 0.001	0.79 (0.77-0.81)	< 0.001
T3	0.65 (0.63,0.66)	< 0.001	0.77 (0.76-0.79)	< 0.001
TT4RI	0.99 (0.99-0.99)	< 0.001	0.99 (0.99-0.99)	< 0.001
T1	Reference		Reference	
T2	0.49 (0.48-0.50)	< 0.001	0.73 (0.71-0.75)	< 0.001
T3	0.55 (0.54-0.56)	< 0.001	0.72 (0.70-0.74)	< 0.001
TFQI	0.72 (0.70-0.73)	< 0.001	0.82 (0.80-0.84)	< 0.001
T1	Reference		Reference	
T2	0.77 (0.75-0.79)	< 0.001	0.92 (0.90-0.94)	< 0.001
T3	0.76 (0.75-0.79)	< 0.001	0.84 (0.82-0.86)	< 0.001
PTFQI	0.60 (0.58-0.61)	< 0.001	0.76 (0.74-0.78)	< 0.001
T1	Reference		Reference	
T2	0.61 (0.59-0.62)	< 0.001	0.83 (0.81-0.85)	< 0.001
T3	0.65 (0.64-0.67)	< 0.001	0.79 (0.77-0.81)	< 0.001
FT3/FT4	1.25 (1.24-1.26)	< 0.001	1.08 (1.07-1.09)	< 0.001
T1	Reference		Reference	
T2	0.56 (0.54-0.57)	< 0.001	0.70 (0.69-0.72)	< 0.001
T3	1.22 (1.19-1.25)	< 0.001	1.00 (0.98-1.02)	0.99

^1^Model 1: adjusted for age, sex;

^2^Model 2: adjusted for age, sex, hypertension, hyperlipidemia, smoking, drinking, current antihypertensive medication, current antilipidemic medication.

### Relationship Between Thyroid Hormone Sensitivity and EBG in Different Sexes

The relationship between thyroid hormone sensitivity and EBG in the different sexes is shown in **Table 3**. Subgroup analyses stratified by sex showed that thyroid hormone sensitivity was significantly associated with EBG in both sexes. After adjusted for age, as a continuous variable, the OR values of males for TSHI, PTFQI, TFQI, and EBG were slightly higher than those of females. These differences remained significant after adjusting for the model (*P <*0.001). Of all the indicators representing central thyroid hormone sensitivity, the PTFQI had the lowest OR value, with females (OR: 0.76; 95%CI: 0.73 to 0.79; *P <*0.001) having a lower PTFQI than males (OR: 0.77; 95%CI: 0.74 to 0.80; *P <*0.001).

**Table 3:** Relationship between thyroid hormone sensitivity and EBG in different sexes.

Sex	Variables	EBG			
		OR (95% CI)^1^	*P-*value	OR (95% CI)^2^	*P-*value
Males	TSHI	0.89 (0.88-0.90)	< 0.001	0.92 (0.91-0.94)	< 0.001
	T1	Reference		Reference	
	T2	0.60 (0.58-0.62)	< 0.001	0.78 (0.76-0.81)	< 0.001
	T3	0.73 (0.71-0.76)	< 0.001	0.78 (0.76-0.81)	< 0.001
	TT4RI	0.99 (0.99-0.99)	< 0.001	0.99 (0.99-0.99)	< 0.001
	T1	Reference		Reference	
	T2	0.55 (0.53-0.56)	< 0.001	0.73 (0.71-0.76)	< 0.001
	T3	0.63 (0.61-0.65)	< 0.001	0.74 (0.72-0.77)	< 0.001
	PTFQI	0.67 (0.64-0.69)	< 0.001	0.77 (0.74-0.80)	< 0.001
	T1	Reference		Reference	
	T2	0.63 (0.61-0.65)	< 0.001	0.79 (0.77-0.82)	< 0.001
	T3	0.73 (0.71-0.76)	< 0.001	0.79 (0.76-0.81)	< 0.001
	TFQI	0.80 (0.77-0.82)	< 0.001	0.83 (0.80-0.86)	< 0.001
	T1	Reference		Reference	
	T2	0.79 (0.76-0.81)	< 0.001	0.90 (0.87-0.93)	< 0.001
	T3	0.86 (0.83-0.88)	< 0.001	0.85 (0.82-0.88)	< 0.001
	FT3/FT4	1.21 (1.20-1.23)	< 0.001	1.08 (1.06-1.09)	< 0.001
	T1	Reference		Reference	
	T2	0.56 (0.54-0.58)	< 0.001	0.68 (0.66-0.71)	< 0.001
	T3	1.09 (1.05-1.12)	< 0.001	0.96 (0.93-0.99)	0.24
Females	TSHI	0.83 (0.82-0.84)	< 0.001	0.91 (0.90-0.92)	< 0.001
	T1	Reference		Reference	
	T2	0.52 (0.51-0.54)	< 0.001	0.80 (0.77-0.83)	< 0.001
	T3	0.57 (0.56,0.59)	< 0.001	0.78 (0.75-0.81)	< 0.001
	TT4RI	0.99 (0.99-0.99)	< 0.001	0.99 (0.99-0.99)	< 0.001
	T1	Reference		Reference	
	T2	0.44 (0.42-0.45)	< 0.001	0.73 (0.70-0.76)	< 0.001
	T3	0.49 (0.47-0.50)	< 0.001	0.71 (0.68-0.73)	< 0.001
	PTFQI	0.54 (0.52-0.56)	< 0.001	0.76 (0.73-0.79)	< 0.001
	T1	Reference		Reference	
	T2	0.59 (0.57-0.61)	< 0.001	0.87 (0.84-0.90)	< 0.001
	T3	0.58 (0.57-0.61)	< 0.001	0.81 (0.78-0.84)	< 0.001
	TFQI	0.64 (0.62-0.67)	< 0.001	0.82 (0.78-0.85)	< 0.001
	T1	Reference		Reference	
	T2	0.75 (0.72-0.77)	< 0.001	0.94 (0.91-0.98)	0.001
	T3	0.68 (0.65-0.70)	< 0.001	0.85 (0.82-0.88)	< 0.001
	FT3/FT4	1.29 (1.27-1.30)	< 0.001	1.07 (1.06-1.09)	< 0.001
	T1	Reference		Reference	
	T2	0.54 (0.53-0.56)	< 0.001	0.72 (0.70-0.75)	< 0.001
	T3	1.35 (1.30-1.39)	< 0.001	1.02 (0.98-1.05)	0.40

^1^Model 1: adjusted for age;

^2^Model 2: adjusted for age, sex, hypertension, hyperlipidemia, smoking, drinking, current antihypertensive medication, current antilipidemic medication.

### Relationship Between Thyroid Hormone Sensitivity and EBG In Different Age Stratifications

The relationship between thyroid hormone sensitivity and EBG in different age stratifications is shown in **Table 4**. After adjusted for sex, as a continuous variable, the OR values of TSHI, PTFQI, and TFQI in patients aged 60 years were slightly higher than those aged > 60 years. These differences remained significant after adjusting for the model (P <0.001). Similar to the sex rating and the age rating, PTFQI also had the lowest OR among all measures representing central thyroid hormone sensitivity, and the PTFQI of patients aged over 60 years (OR: 0.74; 95%CI: 0.72 to 0.77; *P* < 0.001) was lower than that of patients aged 60 years and below (OR: 0.79; 95%CI: 0.76 to 0.83; *P* < 0.001).

**Table 4:** Relationship between thyroid hormone sensitivity and EBG in different age stratification.

Age	Variables	EBG			
		OR (95% CI)^1^	*P*-value	OR (95% CI)^2^	*P*-value
≤60	TSHI	0.88 (0.87-0.89)	< 0.001	0.93 (0.92-0.95)	< 0.001
	T1	Reference		Reference	
	T2	0.58 (0.56-0.60)	< 0.001	0.81 (0.77-0.84)	< 0.001
	T3	0.69 (0.66-0.72)	< 0.001	0.81 (0.78-0.85)	< 0.001
	TT4RI	0.99 (0.99-0.99)	< 0.001	0.99 (0.99-0.99)	< 0.001
	T1	Reference		Reference	
	T2	0.50 (0.48-0.52)	< 0.001	0.73 (0.70-0.76)	< 0.001
	T3	0.59 (0.57-0.61)	< 0.001	0.76 (0.72-0.79)	< 0.001
	PTFQI	0.63 (0.61-0.66)	< 0.001	0.79 (0.76-0.83)	< 0.001
	T1	Reference		Reference	
	T2	0.61 (0.58-0.63)	< 0.001	0.82 (0.78-0.85)	< 0.001
	T3	0.68 (0.66-0.71)	< 0.001	0.81 (0.77-0.84)	< 0.001
	TFQI	0.77 (0.74-0.80)	< 0.001	0.86 (0.82-0.90)	< 0.001
	T1	Reference		Reference	
	T2	0.78 (0.75-0.81)	< 0.001	0.93 (0.89-0.96)	< 0.001
	T3	0.80 (0.77-0.83)	< 0.001	0.86 (0.82-0.90)	< 0.001
	FT3/FT4	1.25 (1.24-1.27)	< 0.001	1.08 (1.06-1.10)	< 0.001
	T1	Reference		Reference	
	T2	0.55 (0.53-0.57)	< 0.001	0.69 (0.66-0.72)	< 0.001
	T3	1.10 (1.05-1.14)	< 0.001	0.93 (0.89-0.97)	0.001
>60	TSHI	0.85 (0.84-0.86)	< 0.001	0.91 (0.90-0.92)	< 0.001
	T1	Reference		Reference	
	T2	0.55 (0.53-0.56)	< 0.001	0.78 (0.75-0.80)	< 0.001
	T3	0.63 (0.61-0.64)	< 0.001	0.76 (0.73-0.78)	< 0.001
	TT4RI	0.99 (0.99-0.99)	< 0.001	0.99 (0.99-0.99)	< 0.001
	T1	Reference		Reference	
	T2	0.48 (0.46-0.49)	< 0.001	0.72 (0.70-0.75)	< 0.001
	T3	0.53 (0.52-0.55)	< 0.001	0.70 (0.68-0.72)	< 0.001
	PTFQI	0.58 (0.56-0.60)	< 0.001	0.74 (0.72-0.77)	< 0.001
	T1	Reference		Reference	
	T2	0.60 (0.59-0.62)	< 0.001	0.83 (0.80-0.86)	< 0.001
	T3	0.64 (0.62-0.66)	< 0.001	0.78 (0.76-0.80)	< 0.001
	TFQI	0.69 (0.67-0.71)	< 0.001	0.80 (0.77-0.83)	< 0.001
	T1	Reference		Reference	
	T2	0.76 (0.74-0.79)	< 0.001	0.92 (0.89-0.94)	< 0.001
	T3	0.74 (0.72-0.77)	< 0.001	0.83 (0.81-0.86)	< 0.001
	FT3/FT4	1.25 (1.24-1.27)	< 0.001	1.08 (1.06-1.09)	< 0.001
	T1	Reference		Reference	
	T2	0.54 (0.53-0.56)	< 0.001	0.70 (0.68-0.72)	< 0.001
	T3	1.28 (1.24-1.31)	< 0.001	1.03 (0.99-1.06)	0.076

^1^Model 1: adjusted for sex;

^2^Model 2: adjusted for age, sex, hypertension, hyperlipidemia, smoking, drinking, current antihypertensive medication, current antilipidemic medication.

### Relationship Between Thyroid Hormone Sensitivity and EBG in Different Sexes and Different Ages

Based on the individual sex and age stratification results, the relationship between thyroid hormone sensitivity and EBG in different sexes and ages was analysed. As shown in **Table 5**, in the unadjusted and adjusted models, all the central thyroid hormone sensitivity indices had lower OR values with EBG in females aged over 60 years old, and the peripheral thyroid sensitivity index had a higher OR with EBG in females aged 60 and below.

**Table 5:** Relationship between thyroid hormone sensitivity and EBG in different sexes and different ages.

Sex	Age	Variables	EBG			
			OR(95% CI)^1^	*P*-value	OR (95% CI)^2^	*P*-value
Males	≤60	TSHI	0.90 (0.89-0.92)	< 0.001	0.94 (0.92-0.96)	< 0.001
		TT4RI	0.99 (0.99-0.99)	< 0.001	0.99 (0.99-1.00)	0.05
		TFQI	0.84 (0.80-0.89)	< 0.001	0.90 (0.85-0.95)	< 0.001
		PTFQI	0.69 (0.65-0.73)	< 0.001	0.81 (0.77-0.86)	< 0.001
		FT3/FT4	1.22 (1.19-1.24)	< 0.001	1.07 (1.05-1.09)	< 0.001
	>60	TSHI	0.88 (0.87-0.89)	< 0.001	0.91 (0.90-0.93)	< 0.001
		TT4RI	0.99 (0.99-0.99)	< 0.001	0.99 (0.99-0.99)	< 0.001
		TFQI	0.77 (0.73-0.80)	< 0.001	0.79 (0.75-0.83)	< 0.001
		PTFQI	0.65 (0.63-0.68)	< 0.001	0.75 (0.71-0.78)	< 0.001
		FT3/FT4	1.22 (1.20-1.24)	< 0.001	1.08 (1.06-1.10)	< 0.001
Females	≤60	TSHI	0.86 (0.84-0.87)	< 0.001	0.93 (0.91-0.95)	< 0.001
		TT4RI	0.99 (0.99-0.99)	< 0.001	0.99 (0.99-0.99)	< 0.001
		TFQI	0.68 (0.63-0.72)	< 0.001	0.82 (0.77-0.89)	< 0.001
		PTFQI	0.56 (0.53-0.60)	< 0.001	0.78 (0.73-0.84)	< 0.001
		FT3/FT4	1.30 (1.27-1.33)	< 0.001	1.09 (1.06-1.12)	< 0.001
	>60	TSHI	0.82 (0.81-0.83)	< 0.001	0.90 (0.89-0.92)	< 0.001
		TT4RI	0.99 (0.99-0.99)	< 0.001	0.99 (0.99-0.99)	< 0.001
		TFQI	0.63 (0.60-0.65)	< 0.001	0.82 (0.78-0.85)	< 0.001
		PTFQI	0.52 (0.50-0.55)	< 0.001	0.75 (0.72-0.79)	< 0.001
		FT3/FT4	1.28 (1.27-1.30)	< 0.001	1.07 (1.05-1.08)	< 0.001

^1^Model 1: unadjusted;

^2^Model 2: adjusted for age, sex, hypertension, hyperlipidemia, smoking, drinking, current antihypertensive medication, current antilipidemic medication.

## Discussion

To our knowledge, this is the first study to evaluate and confirm the relationship between central and peripheral sensitivity to thyroid hormone indicators and EBG risk in a large sample of patients in China with CHD. Our study found that the central thyroid hormone sensitivity indices TSHI, TT4RI, TFQI, and PTFQI were negatively associated with EBG risk in patients with CHD. With a gradual increase in TSHI, TT4RI, and PTFQI, the OR value of EBG also gradually decreased. Peripheral thyroid hormone sensitivity index FT3/FT4 was positively associated with EBG. Finally, most associations were observed in different sexes and age groups when considered separately.

Previous studies have shown that almost two-thirds of patients with cardiovascular disease suffer from abnormal glucose metabolism (30). Due to the various changes of thyroid hormones in patients with CHD (31), the various effects of thyroid hormones in patients with CHD and EBG deserve attention. However, previous studies showed that higher TSH and lower FT4 (16, 32, 33), lower TSH (34), higher FT4 (35), and lower FT3 (16, 34) levels were all associated with the risk of EBG. Therefore, TSH or thyroid hormone levels alone may not be sufficient to explain the relationship between the thyroid system and glycaemic disorders. Given these inconsistencies in previously proposed central thyroid hormone sensitivity indices (TSHI, TT4RI) and peripheral thyroid hormone sensitivity indices FT3/FT4 (28, 36–38), in 2019, Laclaustra et al. proposed a new resistance index of central thyroid hormones: TFQI and PTFQI, which approximates TFQI (29). These new indices may have smaller deviations and will not produce extreme values in cases of thyroid dysfunction, which will help to better explain the different associations between the changes in thyroid hormones and diabetes (29).

Based on the new indices, recent studies have found that the increase in TSHI, TT4RI, and PTFQI was associated with reduced prediabetes risk, and the increased FT3/FT4 ratio was associated with an increased risk of prediabetes (23). Unlike our study, the latter had no significant correlation after adjusting for multiple confounding factors. Moreover, our results also suggest that elevated TFQI is associated with a reduced risk of EBG. Laclaustra et al. reported that the cross-section of the TSHI level was not associated with diabetes (29), which is contrary to our results and conclusion. The reasons for these differences are not clear, but may be attributable to confounding factors, differences in study partitions, and sample sizes. Therefore, further studies are needed to validate and confirm these results.

Several pathways may explain the observed association between the thyroid hormone central resistance index and EBG. Previous studies have shown that thyroid dysfunction can increase insulin resistance in muscle and adipose tissue and reduce glucose transport in muscle cells (39, 40). FT3 may also affect the expression of glucose-secreted insulin and an important protein of lipid metabolism (41); lower FT3 and FT4 levels can promote higher insulin resistance in tissues (42). The change in serum TSH may directly affect metabolic parameters and stimulate leptin secretion (43, 44). It is well known that hepatic glucose output is critical for maintaining fasting glucose homeostasis. Leptin has been shown to stimulate hepatic glucose production *in vivo* and *in vitro* (45). Moreover, the loss of leptin can lead to problems such as overeating, decreased energy expenditure, and severe obesity, which are important risk factors affecting glucose homeostasis (46). Therefore, the sensitivity of central thyroid hormones may change leptin secretion, affect insulin resistance, and lead to a change in glucose metabolism level. However, the exact regulatory mechanism underlying the relationship between central thyroid hormone sensitivity and leptin remains unclear.

Interestingly, our study also found that when used as a reference in the T1 group, the level of peripheral thyroid hormone sensitivity FT3/FT4 was negatively associated with EBG in the T2 group, but significantly positively associated with EBG when used as a continuous variable. Previously, many studies confirmed the positive effects of elevated FT3/FT4 on diabetes, gestational diabetes, obesity-related inflammatory markers, cardiovascular risk, and arterial stiffness markers (38, 47–49). Regarding the result of the negative correlation of FT3/FT4 with EBG in the T2 group, the promotion of peripheral deiodinase activity may increase the level of FT3/FT4 (38), and the inhibition of peripheral deiodinase activity may reduce the basal metabolic rate, which is closely related to the pathogenesis of diabetes (50, 51).

To address sex- and age-specific differences noted in previous studies (35, 52, 53), we analysed the relationship between thyroid hormone sensitivity and diabetes by sex and age, respectively. The results showed that TSHI, PTFQI, and TFQI of females aged > 60 years had lower OR values for EBG risk, while FT3/FT4 had higher OR values. Sex hormones (such as oestrogen and testosterone) can regulate thyroid function, and oestrogen levels affect the development of diabetes, especially after 60 years of age (54–56). The differences in sex hormones may partly explain the sex differences in the relationship between thyroid hormone sensitivity and EBG found in this study. However, since this study did not measure the levels of sex hormones, further research is needed to explore this concept.

## Limitations

There are several limitations to the present study. First, although large scale, this was a cross-sectional study, and causality cannot be inferred. However, the study supports the important hypothesis that adding the examination of thyroid hormone sensitivity levels to the examination of pure thyroid hormone levels may be helpful in assessing the risk of EBG. Second, although we have adjusted for many potential confounding factors, we cannot rule out the possibility that EBG is affected by other lifestyle variables, including iodine supplementation, which is intrinsically related to thyroid hormone levels. Third, this study was conducted among individuals who were Chinese, and racial differences may exist. Fourth, this study did not have a track record of whether participants were undergoing diabetes or thyroid disease-specific treatment. Therefore, well-designed randomized controlled trials are needed to validate these results.

## Conclusion

The decrease in central thyroid hormone indices represents an increase in central thyroid hormone sensitivity. This study showed that thyroid hormone sensitivity is significantly associated with EBG in patients with CHD. This association is higher in females than in males, and the association in those aged over 60 years is higher than that in patients aged 60 years and below. This study provides reliable evidence which will improve the prevention strategies and clinical treatment of EBG in patients with CHD.

## Data Availability Statement

The raw data supporting the conclusions of this article will be made available by the authors, without undue reservation.

## Ethics Statement

This study was approved by the ethics committee of the Tianjin University of Traditional Chinese Medicine (approval number TJUTCM-EC20190008) and registered with the Chinese Clinical Trial Registry on July 14, 2019 (registration number ChiCTR-1900024535) and in ClinicalTrials.gov on July 18, 2019 (registration number NCT04026724). Written informed consent for participation was not required for this study in accordance with the national legislation and the institutional requirements.

## Author Contributions

CY, LL and QX were responsible for the study protocol and statistical analysis. LY and ZL analysed the data together and drafted the article. RY, GP, QC, YH, YL, FL, MM, TY, YW, JS conducted data collection. YZ and SG revised the article critically. All authors revised the article for important intellectual content and approved the article. The authors read and approved the final manuscript. All authors contributed to the article and approved the submitted version.

## Funding

This study was supported by the National Basic Research Program of China (973 project, grant numbers: 2014CB542902).

## Conflict of Interest

The authors declare that the research was conducted in the absence of any commercial or financial relationships that could be construed as a potential conflict of interest.

## Publisher’s Note

All claims expressed in this article are solely those of the authors and do not necessarily represent those of their affiliated organizations, or those of the publisher, the editors and the reviewers. Any product that may be evaluated in this article, or claim that may be made by its manufacturer, is not guaranteed or endorsed by the publisher.

## Abbreviations

CHD, coronary heart disease; CI, confidence interval; DBP, diastolic blood pressure; EBG, elevated blood glucose; FBG, fasting blood glucose; FT3, free triiodothyronine; FT4, free thyroxine; HbA1c, glycated haemoglobin; HDL-C, high-density lipoprotein cholesterol; LDL-C, low-density lipoprotein cholesterol; NGT, normal glucose tolerance; OR, odds ratio; SBP, Systolic blood pressure; SD, standard deviation; TC, total cholesterol; TG, triglyceride; TH, thyroid hormone; TSH, thyroid stimulating hormone.
