# Supplementary material for: Impaired Sensitivity to Thyroid Hormones Is Associated With Elevated Blood Glucose in Coronary Heart Disease
Source: Front Endocrinol (Lausanne). 2022 Jun 15;13:895843. doi: 10.3389/fendo.2022.895843 (PMC9240192; doi:10.3389/fendo.2022.895843)
Supplement: Supplementary file 1 [file DataSheet_1.docx]

**Table S1**. Relationship between thyroid hormone sensitivity indices and prediabetes and diabetes.

**Table S2**. Relationship between gender-specific thyroid hormone sensitivity indices and prediabetes and diabetes.

**Table S3**. Relationship between thyroid hormone sensitivity index and prediabetes and diabetes in different ages and sexes.

**Table S1 Relationship between thyroid hormone sensitivity indices and prediabetes and diabetes**

| **Variables** | **Prediabetes** | | **Diabetes** | |
| --- | --- | --- | --- | --- |
|  | **OR(95% CI)^1^** | **OR(95% CI)^2^** | **OR(95% CI)^1^** | **OR(95% CI)^2^** |
| **TSHI** | **0.87(0.86-0.88)**** | **0.86(0.85-0.87)**** | **0.87(0.86-0.88)**** | **0.83(0.83-0.84)**** |
| ≤1.50 | Reference | Reference | Reference | Reference |
| (1.50-2.49) | 0.61(0.60-0.63)** | 0.61(0.59-0.63)** | 0.52(0.50-0.53)** | 0.51(0.50-0.53)** |
| ≥2.49 | 0.66(0.64-0.68)** | 0.64(0.62-0.66)** | 0.64(0.62-0.66)** | 0.62(0.60-0.63)** |
| **TT4RI** | **0.99(0.99-0.99)**** | **0.99(0.99-0.99)**** | **0.99(0.99-0.99)**** | **0.99(0.99-0.99)**** |
| ≤9.01 | Reference | Reference | Reference | Reference |
| (9.01-25.58) | 0.53(0.52-0.55)** | 0.53(0.52-0.55)** | 0.45(0.44-0.46)** | 0.45(0.45-0.46)** |
| ≥25.58 | 0.59(0.57-0.61)** | 0.57(0.56-0.59)** | 0.52(0.51-0.53)** | 0.50(0.49-0.52)** |
| **TFQI** | **0.72(0.70-0.74)**** | **0.69(0.67-0.71)**** | **0.71(0.70-0.74)**** | **0.68(0.66-0.70)**** |
| ≤-0.23 | Reference | Reference | Reference | Reference |
| (-0.23-0.12) | 0.80(0.77-0.82)** | 0.79(0.77-0.81)** | 0.74(0.72-0.76)** | 0.73(0.71-0.76)** |
| ≥0.12 | 0.76(0.74-0.78)** | 0.73(0.71-0.75)** | 0.77(0.75-0.79)** | 0.73(0.71-0.75)** |
| **PTFQI** | **0.63(0.61-0.65)**** | **0.60(0.59-0.62)**** | **0.57(0.55-0.59)**** | **0.55(0.53-0.56)**** |
| ≤-0.13 | Reference | Reference | Reference | Reference |
| (-0.13-0.24) | 0.65(0.64-0.67)** | 0.65(0.63-0.67)** | 0.57(0.55-0.58)** | 0.62(0.60-0.63)** |
| ≥0.24 | 0.67(0.65-0.69)** | 0.65(0.63-0.67)** | 0.64(0.62-0.66)** | 0.56(0.55-0.58)** |
| **FT3/FT4** | **1.22(1.21-1.24)**** | **1.23(1.22-1.24)**** | **1.28(1.27-1.29)**** | **1.28(1.27-1.30)**** |
| ≤0.29 | Reference | Reference | Reference | Reference |
| (0.29-0.41) | 0.74(0.72-0.76)** | 0.75(0.73-0.77)** | 0.42(0.40-0.43)** | 0.43(0.41-0.44)** |
| ≥0.41 | 1.33(1.29-1.37)** | 1.36(1.32-1.40)** | 1.14(1.11-1.17)** | 1.17(1.14-1.20)** |

^1^Model 1: adjusted for age and sex;

^2^Model 2: adjusted for age, sex, hypertension, hyperlipidemia, smoking, drinking.

Compared with NGT,** *P* < 0.001

**Table S2 Relationship between sex-specific thyroid hormone sensitivity indices and prediabetes and diabetes**

| **Sex** | **Variables** | **Prediabetes** | | **Diabetes** | |
| --- | --- | --- | --- | --- | --- |
|  |  | **OR(95% CI)^1^** | **OR(95% CI)^2^** | **OR(95% CI)^1^** | **OR(95% CI)^2^** |
| Male | TSHI | 0.90(0.89-0.92)****** | 0.89(0.88-0.91)****** | 0.88(0.86-0.89)****** | 0.86(0.85-0.88)****** |
|  | TT4RI | 0.99(0.99-0.99)** | 0.99(0.99-0.99)** | 0.99(0.99-0.99)** | 0.99(0.99-0.99)** |
|  | TFQI | 0.79(0.76-0.82)****** | 0.76(0.73-0.79)****** | 0.80(0.77-0.83)****** | 0.76(0.73-0.79)****** |
|  | PTFQI | 0.70(0.67-0.73)****** | 0.68(0.65-0.71)****** | 0.64(0.61-0.66)****** | 0.61(0.58-0.64)****** |
|  | FT3/FT4 | 1.18(1.16-1.20)****** | 1.19(1.17-1.21)****** | 1.24(1.23-1.26)****** | 1.25(1.23-1.27)****** |
| Female | TSHI | 0.84(0.83-0.85)****** | 0.83(0.82-0.84)****** | 0.82(0.81-0.83)****** | 0.81(0.80-0.82)****** |
|  | TT4RI | 0.99(0.99-0.99)** | 0.99(0.99-0.99)** | 0.99(0.99-0.99)** | 0.99(0.99-0.99)** |
|  | TFQI | 0.65(0.62-0.68)****** | 0.62(0.59-0.65)****** | 0.64(0.61-0.66)****** | 0.60(0.58-0.63)****** |
|  | PTFQI | 0.56(0.54-0.59)****** | 0.54(0.52-0.57)****** | 0.51(0.49-0.54)****** | 0.49(0.47-0.51)****** |
|  | FT3/FT4 | 1.26(1.24-1.28)****** | 1.27(1.25-1.29)****** | 1.31(1.29-1.33)****** | 1.32(1.30-1.33)****** |

^1^Model 1: adjusted for age;

^2^Model 2: adjusted for age, hypertension, hyperlipidemia, smoking, drinking.

Compared with NGT,** *P* < 0.001

**Table S3 Relationship between thyroid hormone sensitivity and prediabetes and diabetes in different ages and sexes**

| **Age** | **Variables** | **Prediabetes** | | **Diabetes** | |
| --- | --- | --- | --- | --- | --- |
|  |  | **OR(95% CI)^1^** | **OR(95% CI)^2^** | **OR(95% CI)^1^** | **OR(95% CI)^2^** |
| ≤60 | TSHI | 0.88(0.87-0.90)****** | 0.87(0.86-0.89)****** | 0.88(0.87-0.90)****** | 0.87(0.86-0.89)****** |
|  | TT4RI | 0.99(0.99-0.99)** | 0.99(0.99-0.99)** | 0.99(0.99-0.99)** | 0.99(0.99-0.99)** |
|  | TFQI | 0.73(0.69-0.77)****** | 0.71(0.67-0.75)****** | 0.81(0.77-0.85)****** | 0.78(0.74-0.83)****** |
|  | PTFQI | 0.63(0.60-0.67)****** | 0.61(0.58-0.65)****** | 0.64(0.60-0.67)****** | 0.62(0.59-0.66)****** |
|  | FT3/FT4 | 1.25(1.23-1.27)****** | 1.25(1.23-1.28)****** | 1.26(1.23-1.28)****** | 1.25(1.23-1.28)****** |
| ＞60 | TSHI | 0.86(0.85-0.87)****** | 0.85(0.84-0.86)****** | 0.93(0.90-0.95)****** | 0.82(0.81-0.83)****** |
|  | TT4RI | 0.99(0.99-0.99)** | 0.99(0.99-0.99)** | 0.99(0.99-0.99)** | 0.99(0.99-0.99)** |
|  | TFQI | 0.71(0.68-0.74)****** | 0.68(0.65-0.70)****** | 0.67(0.65-0.70)****** | 0.63(0.61-0.66)****** |
|  | PTFQI | 0.62(0.60-0.65)****** | 0.60(0.58-0.62)****** | 0.54(0.52-0.56)****** | 0.51(0.49-0.53)****** |
|  | FT3/FT4 | 1.21(1.20-1.23)****** | 1.22(1.20-1.24)****** | 1.29(1.27-1.31)****** | 1.30(1.28-1.31)****** |

^1^Model 1: adjusted for sex;

^2^Model 2: adjusted for sex, hypertension, hyperlipidemia, smoking, drinking.

Compared with NGT,** *P* < 0.001
